# Resting metabolic rate and energy efficiency in response to an intensive 84-day combat-swimmer training in the German Armed Forces

**DOI:** 10.1007/s00421-024-05659-0

**Published:** 2024-11-25

**Authors:** Tony H. Richter, Wiebke Braun, Lorenz Scheit, Jan Schröder, Rüdiger Reer, Volker Harth, Katrin Bender, Andreas Koch, Anja Bosy-Westphal, Manfred J. Müller

**Affiliations:** 1 Bundeswehr Joint Medical Service, Regional Medical Support Command, Sanitätsunterstützungszentrum Munster, Sanitätsversorgungszentrum Faßberg, Faßberg, Germany; 2 Institute of Human Nutrition and Food Science, Kiel University, Kiel, Germany; 3 Clinic I - Internal Medicine, Bundeswehr Hospital Hamburg, Hamburg, Germany; 4 Faculty of Psychology and Human Movement Science, Department of Sports Medicine, Institute for Human Movement Science, University of Hamburg, Hamburg, Germany; 5 Institute for Occupational and Maritime Medicine (ZfAM), University Medical Center Hamburg-Eppendorf (UKE), Hamburg, Germany; 6 Section for Maritime Medicine, German Naval Medical Institute and Christian-Albrechts-University Kiel, Kiel, Germany

**Keywords:** Energy expenditure, Body composition, Combat training, Exercise, Physical activity, Energy constraint

## Abstract

**Purpose:**

According to the ‘constrained model’, there are compensations in resting metabolic rate (RMR) at high levels of physical activity (PA). Here, we have used a standardized combat-swimmer training protocol (CST) to investigate whether changes in RMR (i) confirm the ‘constraint model’, and (ii) differ between successful participants and dropouts.

**Methods:**

Controlled 84d CST in 44 male soldiers with 13 finally successful. Fat mass (FM) and fat-free mass (FFM) were measured using Quantitative Magnetic Resonance. RMR was assessed by indirect calorimetry, VO_2max_, and work efficiency by treadmill spiroergometry. Plasma levels of thyroid hormones, testosterone, and cortisol were analysed by standard laboratory methods.

**Results:**

CST increased VO_2max_ (+ 6.9%) and exercise efficiency at low workloads of 10 and 12 km/h (+ 8.7 and + 6.5%; both *p* < 0.05). As energy balance was moderately negative (−356 ± 383 kcal/d), FFM and FM decreased (−2 and −16%; both *p* < 0.05). There was a considerable inter-individual variance but no change in in the mean values of RMR and RMR_adjFFM_. RMR_adjFFM_ before CST had a negative association with its decrease with CST (*p* < 0.005). Concomitantly, plasma hormone levels were unchanged. When compared with dropouts, successful participants had a higher VO_2max_ at baseline (5.2 ± 0.6 vs. 4.9 ± 04 l/min; *p* < 0.05) that increased with CST (+ 4.4 vs. −0.4%; *p* < 0.05) at similar changes in body composition and energy balance.

**Conclusion:**

While CST increased VO_2max_ and exercise efficiency as a compensation, there was an inter-individual variance in exercise-related compensation of RMR with no differences between ‘completers’ and ‘non-completers’.

**Trial registration** DRKS00018850, November 27, 2019.

## Introduction

Total energy expenditure (TEE) is the energy (i) allocated to growth, reproduction, and body weight maintenance (i.e., resting metabolic rate, RMR, in a thermoneutral environment and a post-absorptive state), (ii) related to digestion and metabolism of food, as well as (iii) expended on spontaneous and planned physical activity (PA; i.e., activity energy expenditure, AEE). AEE is the most variable component of TEE across and within populations. Among adults, body size and composition, age, sex, and PA explain about 60–70% of the variance in TEE (Pontzer et al. 2021). In addition, inter- and intra-individual variations in energy expenditure (EE) reflect the plasticity and flexibility of EE and trade-offs of energy between different organs and tissues which are sensitive to temperature, food availability, PA, workload, and diseases. Metabolic adaptation to high PA includes decreases in RMR (i.e., in essential metabolic functions) and increases in exercise efficiency at increases in activity energy expenditure (AEE; Flanagan et al. 2024).

In humans, TEE may increase linearly with increases in PA (i.e., the ‘additive model’ of energy budgets suggesting an unchanged energy allocation to body maintenance and reproduction at high PA). By contrast, the ‘constrained model’ of energy budgets assumes an asymptotical increase in TEE with increasing PA resulting in adaptions of RMR reflecting a reduced allocation of energy to body maintenance, growth, and reproduction. The ‘constrained model’ implies that at high PA, TEE has reached an upper limit where RMR decreases (with a decrease in size- and tissue-specific metabolic rates of organs and tissues) giving the capacity for AEE to increase (Pontzer et al. 2016). Evidence of trade-offs between AEE and TEE on other domains has been found in observational studies on children (Urlacher et al. 2019) and adult populations (Careau et al. 2021). The ‘constraint model’ fundamentally questions current concepts in nutritional science, clinical nutrition, and sports medicine.

Exercise-related energy compensation may be similar to the previous finding that RMR decreases in response to caloric restriction which is associated with decreases in fat-free mass (FFM) and fat mass (FM) as well as in the plasma concentrations of fT3, leptin, and testosterone, while cortisol levels increased (Nindl et al. 2007; Müller and Bosy-Westphal 2013; Müller et al. 2015). The decrease in RMR is in part independent of the decrease in FFM reflecting a mass-independent decrease in cellular respiration which is described as ‘adaptive thermogenesis’ (Müller et al. 2015, 2016a, 2021, 2023a). In addition, with weight loss there is a 10–20% increase in the mitochondrial efficiency (i.e., the ratio of oxygen consumed per ATP produced) in skeletal muscles (Rosenbaum and Leibel 2010; Müller et al. 2015). These phenomena add to the variability of metabolic adaptation which includes adaptations in RMR and in the non-resting compartment of EE (Müller et al. 2023b). However, since a negative energy balance during an intensive training protocol will result in a decrease in FM and, possibly, FFM, this may impact trade-offs of energy between different organs and tissues and, thus, the ‘constraint model’.

Studies supporting the ‘constraint model’ (i) were mostly observational in free living subjects at different levels of PA, (ii) did not take into account the effects of a negative energy balance (due to excessive exercise with at the same time insufficient energy intake) and its effect on possible hormonal determinants of EE, and (iii) had some methodological and statistical limitations (Gonzalez et al. 2023). Recently, two publications have put the ‘constraint model’ into perspective. First, there was a ‘constrained energy expenditure’ under conditions of an energy deficit (due to an inadequate energy intake) only (Willis et al. 2022). A reduced energy intake and, thus, a catabolic state was observed within 2 weeks after the start of a military training protocol and had a negative impact on soldiers' performance (Nindl et al. 2007). And second, there was no evidence for metabolic adaptations in sleep or resting energy expenditures in response to a controlled 24-week exercise intervention (Flanagan et al 2024).

In summary, possible adaptations of RMR in response to a high PA resulting from an extensive training program remain unclear. We hypothesized that (i) extensive training results in a mass-independent decrease in RMR (which would suggest ‘exercise energy compensation’) and (ii) unsuccessful participants show an impaired metabolic and hormonal adaptation together with detrimental changes in body composition.

## Methods

### Study protocol

Soldiers have special physical demands and special physical stresses. As “tactical athletes”, soldiers benefit from systematic training to optimize their performance and to prevent injuries or illnesses (Sell et al. 2010). Thus, sports medical and nutritional care of soldiers with special stresses is obligatory. For the Bundeswehr in general and, in this context, for the German Navy’s Special Forces Command (Kommando Spezialkräfte der Marine, KSM) in particular, it is relevant that (i) the soldiers are optimally trained, (ii) skills and potential of the soldiers are recognised, promoted in the sense of ‘human performance optimisation’ (Travis and Brown 2023) and optimally utilised to meet the constantly growing and varying challenges the KSM is confronted with.

The metabolic effects of training of German Navy’s Special Forces Command combat-swimmers have not been investigated before. Our ‘Combat-swimmer training’ (CST) study was therefore launched as prospective observational study to investigate how combat-swimmer students are challenged mentally and physically during the first twelve weeks of the training program and how they adapt to it metabolically. The aim of the study was, among other things, to investigate if the participants who dropped out within the first twelve weeks of training program differed in their metabolic and endocrine adaptations from the successful participants. In addition, the study protocol intended to identify parameters that allow an early differentiation between successful and unsuccessful participants, to potentially reduce the number of unsuccessful participants by targeted intervention during the training program.

CST (registered with DRKS, No. DRKS00018850) is a prospective observational study with a pre/post design. Combat-swimmer trainees were monitored before (T0) and during an 11-week phase of training with 6-week exercise preconditioning (“EP”, i.e., phase T0 to T1) the subsequent 5-week indoor phase training phase with a focus on specific skills and stress responses ("SSS", i.e., from T1 to T2) and one following week of examination phase. The study protocol complied with the statutes of the Declaration of Helsinki and was approved by the ethics committee of the Medical Faculty of the Christian-Albrechts-Universität, Kiel (D 423/19).

The study was conducted between November 2021 and June 2023. All participants had been investigated during the first and last week of “EP” and the week after “SSS”.

### Research questions

We have addressed two research questions:

- Is there a decrease in RMR in response to CST?
- Do the metabolic and endocrine responses to CST allow a differentiation between successful and unsuccessful participants (i.e., ‘completers’ vs. ‘non-completers’?

### Participants

Study participants were recruited from soldiers who had started the CST and volunteered to take part. All subjects provided informed written consent before participation. Participation could be stopped at any time without giving reasons and without negative consequences. There was no financial benefit or any other form of remuneration or favour for participating in the study. As only male candidates have qualified for CST to date, the study is limited to male volunteers of legal age with a valid diving medical certificate in accordance with Bundeswehr regulation C1-258/03000 for TUKV TA3 (diver, submarine, combat-swimmer suitability). Failure to fulfil the above conditions was considered an exclusion criterion.

Participation in the study ended after T1 (i.e., at the end of EP, these subjects were called ‘exercise completers’) or T2 (i.e., after EP + SSS, these subjects were called 'full completers’) were reached or in the case that the soldiers had to end the training due to illness or injury (health reasons, e.g., SARS-COV 2 and other viral or bacterial infections, barotrauma, and severe bruises) or did not fulfil the requirements of the training or if they wanted to end their participation for personal reasons.

The baseline data (at T0) on body composition, REE, fitness and hormones of all participants and stratified according to ‘non-eligible soldiers’, ‘exercise completers’, and ‘full completers’ are presented in Tables 1 and 2. Forty-four soldiers had been assessed for eligibility in the study, but only 28 soldiers met the inclusion criteria and were enrolled. 16 soldiers cancelled the program during the first six weeks of the CST, six left for personal reasons, three had to end participation for health reasons, and six had to drop out due to insufficient performance. Sixteen soldiers have completed “EP” (i.e., at T1; ‘exercise completers’, mean age 25.7 ± 4.1 years), while 13 soldiers completed the entire program until T2 (i.e., ‘full completers’, mean age 25.23 ± 2.83 years, Table 2).

**Table 1:** Characterization of all participants before the start of the combat swimmer training (CST)

*N* = 44	Mean	SD	Range
Basal characteristics			
Age (y)	25.75	± 3.02	20–36
Height (cm)	182	± 7	170–197
Weight (kg)	82.62	± 8.41	69.50–110.20
BMI (kg/m^2^)	24.87	± 1.68	20.60–28.40
FMI (kg/m^2^)	3.02	± 0.98	0.61–5.40
FFMI (kg/m^2^)	19.72	± 1.80	15.80–25.20
MM (kg)	31.24	± 3.28	25.37–40.41
Aerobic fitness			
VO_2max_ (ml/min)	5085^a^	± 514^a^	4198–6302^a^
VO_2max_ (ml/min/kg)	62^a^	± 5^a^	48–70^a^
Resting energy expenditure			
REE (kcal/d)	2139.07	± 277.54	1597–2626
REEadj (kcal/d)	1855.89	± 241.84	1368–2490
REEpred (kcal/d)	1939.99	± 141.29	1712–2324
REEm–REEp (kcal/d)	199	± 213.55	−291–765
Respiratory exchange ratio	0.78	± 0.08	0.70–1.10

*BMI* body mass index; *FMI* fat mass index; *FFMI* fat free mass index; *MM* muscle mass; *VO*_*2max*_ maximum volume of oxygen consumed; *REE* resting energy expenditure; *REEadj* REE adjusted for FFM using regression analysis; *REEpred* predicted REE acc. to Harris and Benedict; *REEm − REEp* measured REE minus predicted REE acc. to Harris and Benedict; ^a^*n* = 35

**Table 2:** Characterization of completers and dropouts before the start of the combat swimmer training (CST)

*N* = 44	Full completers (*n* = 13)	Exercise completers (*n* = 15)	Non eligible soldiers (*n* = 16)
Body weight and body composition			
Height (cm)	183 ± 6	182 ± 9	182 ± 6
Weight (kg)	83.04 ± 5.81	85.94 ± 10.50*	78.83 ± 6.56
BMI (kg/m^2^)	24.79 ± 1.42	26.01 ± 1.43*	23.88 ± 1.49
FMI (kg/m^2^)	2.58 ± 0.69	3.20 ± 1.30	3.22 ± 0.79
FFMI (kg/m^2^)	19.96 ± 2.27	20.67 ± 1.13*	18.63 ± 1.42
MM (kg)	31.73 ± 2.45	29.33 ± 1.98*	32.84 ± 3.84
Aerobic fitness			
VO_2max_ (ml/min)	5151 ± 561^a^	5244 ± 550^b^	4860 ± 374^c^
VO_2max_ (ml/min/kg)	62 ± 7^a^	61 ± 4^b^	63 ± 4^c^
Resting energy expenditure			
REE (kcal/d)	2168 ± 288	2197 ± 305	2050 ± 224
REEadj (kcal/d)	1858 ± 283	1854 ± 259	1858 ± 203
REEpred (kcal/d)	1954 ± 110	1990 ± 177	1882 ± 111
REEm–REEp (kcal/d)	214 ± 234	220 ± 216	167 ± 187
Respiratory exchange ratio	0.79 ± 0.10	0.79 ± 0.08	0.76 ± 0.05
Work efficiency			
EE 10 km/h (kcal/min)	15.60 ± 2.72^a^	15.24 ± 2.20^b^	13.44 ± 1.06^c#^
EE 10 km/h (kcal/min/kg)	0.19 ± 0.02^a^	0.18 ± 0.02^b^	0.17 ± 0.01^c^
EE 10 km/h (kcal/min, adjFFM)	15.31 ± 2.14^a^	14.73 ± 1.80^b^	14.25 ± 0.98^c^
EE 12 km/h (kcal/min)	19.03 ± 2.46^a^	18.83 ± 2.26^b^	17.09 ± 1.30^c^
EE 12 km/h (kcal/min/kg)	0.23 ± 0.02^a^	0.22 ± 0.01^b^	0.22 ± 0.02^c^
EE 12 km/h (kcal/min, adjFFM)	18.66 ± 2.09^a^	18.19 ± 1.45^b^	18.09 ± 1.18^c^
EE 14 km/h (kcal/min)	22.25 ± 2.58^a^	21.92 ± 2.75^b^	20.02 ± 1.33^c^
EE 14 km/h (kcal/min/kg)	0.27 ± 0.02^a^	0.26 ± 0.01^b^	0.26 ± 0.01^c^
EE 14 km/h (kcal/min, adjFFM)	21.82 ± 2.21^a^	21.16 ± 1.76^b^	21.19 ± 1.00^c^
EE 16 km/h (kcal/min)	24.19 ± 3.03^a^	24.83 ± 2.98^b^	22.70 ± 1.60^c^
EE 16 km/h (kcal/min/kg)	0.29 ± 0.03^a^	0.29 ± 0.02^b^	0.29 ± 0.01^c^
EE 16 km/h (kcal/min, adjFFM)	23.79 ± 3.17^a^	24.14 ± 1.95^b^	23.79 ± 1.24^c^
Hormones			
fT3 (ng/dl)	0.37 ± 0.05	0.39 ± 0.05	0.38 ± 0.03
fT4 (ng/dl)	1.26 ± 0.15	1.40 ± 0.18	1.39 ± 0.17
TSH (mU/l)	1.87 ± 0.33	1.81 ± 0.74	1.74 ± 0.65
Testosterone (nmol/l)	20 ± 7	21 ± 8	19 ± 5^d^
Cortisol (µmol/l)	0.18 ± 0.05	0.17 ± 0.05	0.20 ± 0.06
Testosterone/cortisol (nmol/l/µmol/l)	132.97 ± 95.59	142.14 ± 84.12	108.71 ± 58.61^d^

Data are presented as mean ± standard deviation

*BMI* body mass index; *FMI* fat mass index; *FFMI* fat free mass index; *MM* muscle mass; *VO*_*2max*_ maximum Volume of Oxygen consumed; *REE* resting energy expenditure; *REEadj* REE adjusted for FFM using regression analysis; *REEpred* predicted REE acc. to Harris and Benedict; *REEm − REEp* measured REE minus predicted REE acc. to Harris and Benedict; *EE* energy expenditure; (kcal/min, adjFFM): energy expenditure adjusted for FFM using regression analysis; *fT3* triiodothyronine; *fT4* thyroxine; *TSH* thyroid stimulating hormone

^*^Sign. difference between Exercise completers vs. Non eligible soldiers, *p* < 0.05; ^#^sign difference between Full completers vs. Non eligible soldiers, *p* < 0.05; ^a^*n* = 10; ^b^*n* = 13; ^c^*n* = 12; ^d^*n* = 14

### Training protocol

CST started with “EP”. During EP, the soldiers were supervised by instructors and supported and challenged both collectively and individually. Each training week comprised 5 days with regular physical exertion and 2 days at the soldiers’ own disposal with no planned exercise sessions. EP included daily high-frequency aerobic training, workloads in the anaerobic range, and regenerative training content, and the daily structure did not include any longer breaks from workload apart from the lunch break. Types of exercise during this phase included endurance training in the form of swimming, diving, apnoea diving, running training indoors and outdoors, strength training, rescue, and recovery exercises, first aid, coordination, and psychological skills training.

Due to the adaptation of the workload during training to the collective but also the individual requirements of the combat swimming soldiers, it is not possible to make a uniform statement on the intensity of the workload during “EP” across the four training programs under consideration. The aim of the sports training phase was to prepare the study participants for the following challenges in the best feasible way within the scope of their potential.

The subsequent “SSS” module also included the burdens, which stressed the combat swimming soldiers in the aerobic, anaerobic, and regenerative stress ranges. In addition to the stresses of “EP”, the “SSS” module also included psychological stressors, such as sleep deprivation, unplanned exertion, night alarms, and changing exertion intensities. There are standardized specifications “SSS”, within the framework of which all combat swimming soldiers were subjected to the same type and intensity of stress. Therefore, an average value of 18,894 MET min/week (range between 1740 and 2739 MET min/day) could be calculated for the SSS module.

### Resting metabolic rate (RMR)

RMR was assessed by indirect calorimetry (IC) using the Vmax Spectra 29n, SensorMedics® (Viasys Healthcare, Bilthoven, The Netherlands, software: Vmax, version 12-1a) and the Quark RMR (COSMED, Rome, Italy, software: Quark RMR, PFT CPET Suite, version 9.1b). Oxygen consumption (VO_2_) and carbon dioxide production (VCO_2_) were measured after prior calibration and regular calibrations during the measurement according to the manufacturer's specifications. Alcohol burning tests were performed as a post-calorimetric test, and any deviation in VO_2_ and VCO_2_ from the theoretical value was used for device-specific corrections (i.e., between −1 and 4%). The subjects’ heads were placed under a transparent ventilated hood. VO_2_ and VCO_2_ were analysed continuously, measured values were averaged every 20 s (Viasys) or every 5 s (COSMED). Measurements were performed early in the morning after an overnight fast under steady state conditions. During the measurement, subjects were in a lying position, awake and, apart from normal breathing movements, did not move. Gas exchange measurements were performed for 40 min, and the data obtained during the first 10 min were discarded. The precision of the IC measurements was 4.4–6.5% (Bader et al. 2005). RMR was calculated using the Weir formula (Weir 1949)

$${\text{RMR}}\;{\text{[kcal/d]}} = \left( {3.9 \times {\text{VO}}_{2} \;{\text{[l/min]}} + 1.1 \times {\text{VCO}}_{2} \;[{\text{l/min}}]} \right) \times 1440.$$

RMR was then adjusted for FFM and the change in FFM (Bosy-Westphal et al. 2013)

$${\text{RMR}}_{{{\text{adjusted}}}} \;\left[ {{\text{kcal}}/{\text{d}}} \right] = {\text{RMR}}_{{{\text{measured}}}} \;\left[ {{\text{kcal}}/{\text{d}}} \right] + \left( {\left( {{52}.{94} - {\text{FFM}}\;\left[ {{\text{kg}}} \right]} \right) \times {22},{762}} \right).$$

In addition, RMR was predicted (‘RMRpred’) according to Harris and Benedict’s algorithm (Harris and Benedict 1918). The respiratory exchange ratio was calculated as VCO_2_/VO_2_.

### Aerobic fitness and work efficiency

Tests were performed on a treadmill spiroergometry (QUARK RMR, COSMED, Rome, Italy; Software: Quark RMR, PFT CPET Suite, version 9.1b). The protocol to assess work efficiency started at 8 km/h with a 1° incline and was increased by 2 km/h every 3 min and monitored for 6 min after the end of the exercise. To reach recovery, the subsequent walking speed was 4 km/h. During spiroergometry, VO_2_, VCO_2_ and heart rate (HR) were measured continuously using a mask system, EE and VCO_2_/VO_2_ were calculated. As the EE adapts to changes in FFM, the data were adjusted to FFM using regression analysis (“EE × km/h (kcal/min, FFM_adjusted_)”) (see above).

### Weight status and body composition

Height and weight were measured with a stadiometer with an accuracy of 1 mm and a calibrated scale (with an accuracy of 10 g; seca 285, seca GmbH & co Hamburg, Germany). Subjects were weighed with an empty bladder, fasting, and in underwear.

Since combat-swimmers are heavy performers, body weight and BMI cannot characterize the functional component of the body, i.e., lean mass or FFM (Potter et al. 2022). Thus, the assessment of body composition was an essential part of physical characterization of the participants of CST. FM was measured in kg using the Quantitative Magnetic Resonance method (QMR) with the EchoMRI™-Adult Human NMR/MRI Body Composition Analyser from Echo Medical Systems, LLC, Houston, USA (for further details of the method, see Bosy-Westphal and Müller 2015). FFM was then calculated by subtracting FM from body weight. QMR assesses FM with high accuracy of 0.2 kg and precision of 0.7% (Napolitano et al. 2008; Bosy-Westphal and Müller 2015; Müller et al 2016b). QMR was calibrated daily following the manufacturer's instructions, at a constant room temperature of between 22 and 23 °C. One measurement run consisted of three consecutive measurements, and the results were averaged. The measurement duration per subject was 6 min.

For inter-individual comparisons, FM and FFM were adjusted for height squared and presented as fat mass index (FMI) and fat-free mass index (FFMI).

Skeletal muscle mass (MM) was calculated from FFM and FM using a formula based on a database of 318 men aged between 18 and 86 years where muscle mass was precisely determined using whole-body MRI scans (Enderle et al. 2023)

$${\text{MM}}\;{\text{(kg)}} = - 0.{5}0{6} + \left( {0.{486} \times {\text{FFM}}\;{\text{(kg)}}} \right) + \left( {0.0{97} \times {\text{FM}}\;{\text{(kg)}}} \right) + \left( { - 0.0{39} \times {\text{age}}\;{\text{(years)}}} \right).$$

### Energy balance

Energy balance (EB) was calculated from changes in body composition (see Müller et al. 2023a)

$${\text{EB}}\;{\text{(kcal}}/{\text{d)}} = \left[ {\left( {\Delta {\text{FM}}\;{\text{(kg)}}* \, x} \right) + \left( {\Delta {\text{FFM}}\;{\text{(kg)}}*y} \right)} \right]/{\text{d}},$$

with *x* = 13,100 kcal/kg for an increase in FM and −9300 kcal/kg for a decrease in FM. In the case of an increase in FFM, *y* = 2200 kcal/kg and in the case of a decrease in FFM *y* = −1100 kcal/kg (taking into account that the synthesis of FM and FFM is more energy-intensive than their actual energy density; Hall and Guo 2017).

### Plasma hormone concentrations

Blood samples were taken in the morning after an overnight fast. Routine laboratory parameters, hormones, and substrates were analysed with the use of standard laboratory techniques (Bundeswehrkrankenhaus Hamburg, Department of Laboratory Medicine; University Medical Center Hamburg-Eppendorf, Institute of Clinical Chemistry and Laboratory Medicine). Normal ranges, cortisone (133–537 nmol/l), testosterone (12.14–39.87 nmol/l), TSH (0.3–3.5 mU/l), fT3 (0.3–0.6 ng/dl) and fT4 (0.5–2.3 ng/dl) (Roche Cobas e411, ECLIA).

### Statistics

Statistical analysis was conducted as inter- and intra-individual comparisons of data before and after intervention (i.e., ANOVA, ANOVA with repeated measures and post hoc Bonferroni as well as Greenhouse–Geisser correction, Mann–Whitney *U* test, and pairwise comparisons) using IBM SPSS Statistics (version 28) as statistical program.

In an ANOVA with post hoc Bonferroni correction, the parameters of all 44 subjects at time T0 (start of study) were compared, analysing whether there was an initial difference between successful soldiers who reached T2 and participants who were not eligible according to the baseline examination or dropped out after T1 (end of EP, start of SSS) or who reached T2 (end of SSS). This comparison revealed a difference between the number of subjects and the analysed parameters of metabolic and hormonal adaptation. In these nine cases, bicycle spiroergometry was performed instead of treadmill spiroergometry. As the performance data from these different measurement methods are not directly comparable (Price et al. 2022; Wiecha et al. 2022), these data were excluded from the analysis. Due to limited blood sample volume to determine all parameters, there were two missing values of the testosterone measurements.

A Mann–Whitney test and Welch test were performed to analyse the inter-individual adaption to compare the subjects who reached T2 with the subjects who dropped out at T1.

A repeated-measures ANOVA (with post hoc Bonferroni and Greenhouse–Geisser correction) was performed to analyse the adaption of basal characteristics, energy expenditure, and hormone parameters in both, the 15 ‘exercise completers’ and the 13 ‘full completers’.

## Results

### Is there a decrease in RMR in response to CST?

**Fig. 1:**
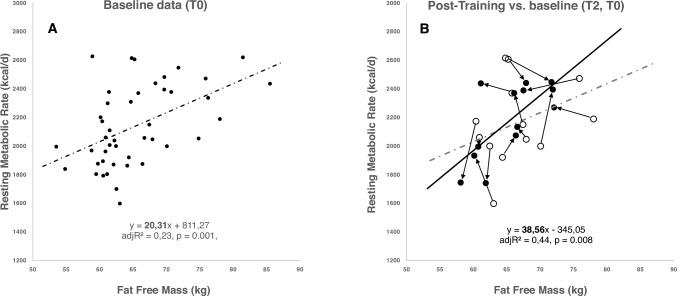
Linear regression analysis of resting energy expenditure (RMR) on fat-free mass (FFM). **A** for the total study population at baseline (T0; *n* = 44). **B** linear regression analysis RMR on FFM in the group of ‘full completers’ (T2; post training T2, *n* = 13) and the change of RMR from T0 to T2. Circles: participants at T0. Black dots: participants at T2

**Fig. 2:**
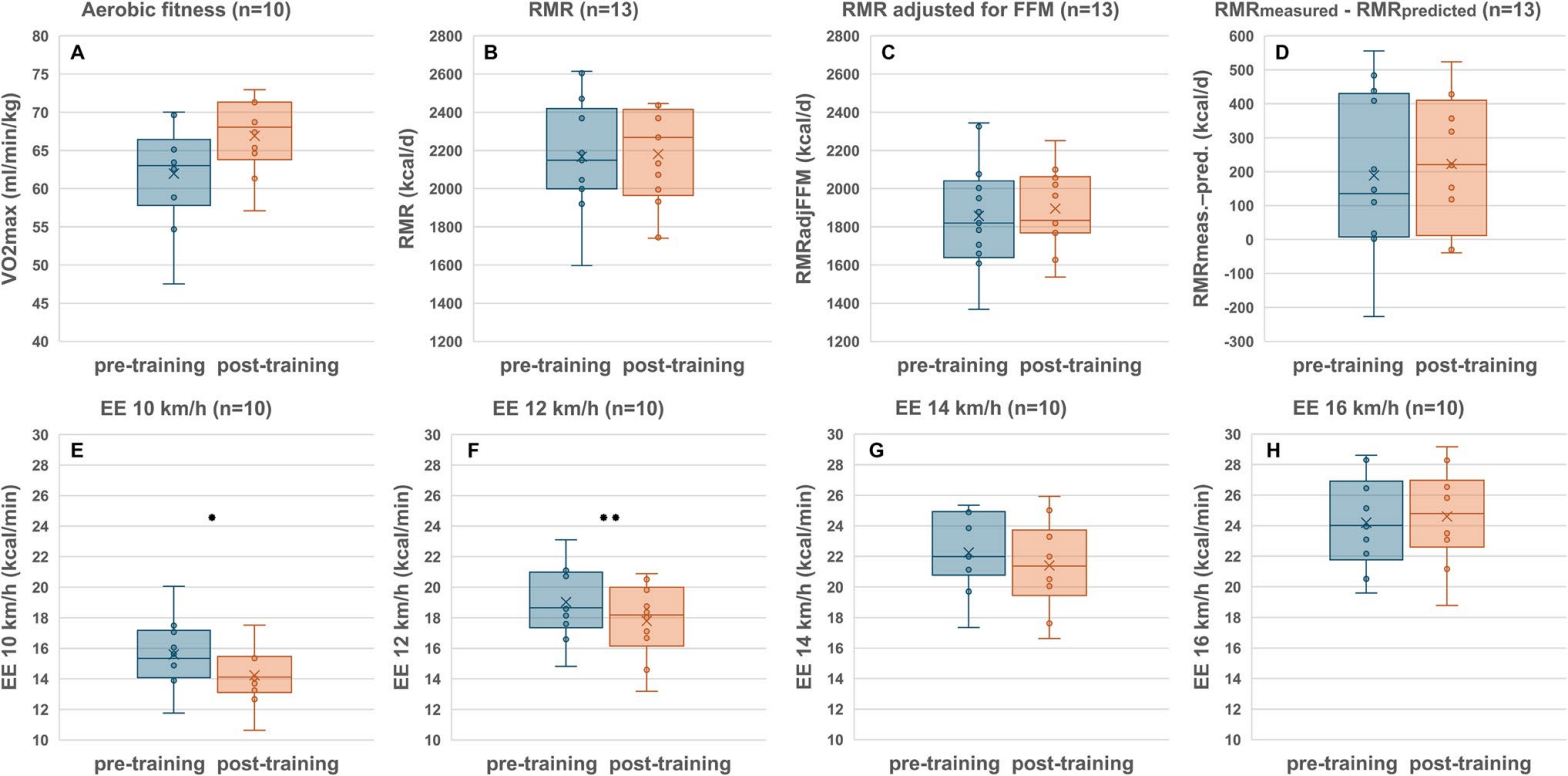
Metabolic and energetic adaptation in the group of ‘full completers’ (*n* = 13). Data are shown as initial state at T0 and at the end of the training protocol (T2). **A** aerobic fitness (VO_2max_), RMR; **B** resting metabolic rate; **C** RMR adjusted for FFM; **D** difference between measured and predicted RMR (with prediction according to the Harris–Benedict algorithm); **E–H** work efficiency at different workloads (as indicated). Significant differences as indicated as *p < 0.05; **p < 0.01

CST reduced body weight, FMI, and muscle mass (Table 3). Energy balance was slightly but not significantly negative (Table 3). While CST increased physical fitness (Fig. 2A), there was no decrease in RMR and no mass-independent adaption of RMR (as indicated by the difference between measured and predicted RMR as well as changes in RMR adjusted for changes in FFM; Figs. 1, 2B–D). CST increased muscle work efficiency at low workload (i.e., EE 10 km/h and EE 12 km/h; Fig. 2E, H). CST did not affect the plasma concentrations of hormones involved in metabolic adaptation: fT3, testosterone, and cortisol levels were unchanged with training as was the case for plasma testosterone concentrations (Table 3).

**Table 3:** T2T0-Changes in body weight and body composition as well as metabolic and endocrine adaptations to combat swimmer training in the group of ‘Full completers’

N = 13	Baseline (T0)	End of training protocol (T2)	∆ T2 − T0
Body weight and body composition			
Weight (kg)	83.04 ± 5.81	81.86 ± 5.85	−1.18 ± 1.40*
BMI (kg/m^2^)	24.79 ± 1.42	24.43 ± 1.40	−0.36 ± 0.44*
FMI (kg/m^2^)	2.58 ± 0.69	2.16 ± 0.53	−0.42 ± 0.40**
FFMI (kg/m^2^)	19.96 ± 2.27	19.59 ± 1.57	−0.37 ± 1.21
MM (kg)	31.74 ± 2.55	31.07 ± 2.36	−0.67 ± 1.93
EB (kcal/d)			−356 ± 383
Respiratory exchange ratio	0.79 ± 0.10	0.86 ± 0.10	0.06 ± 0.11
Hormones			
fT3 (ng/dl)	0.37 ± 0.05	0.36 ± 0.04^a^	−0.01 ± 0.03^a^
fT4 (ng/dl)	1.26 ± 0.15	1.29 ± 0.12^a^	0.01 ± 0.11^a^
TSH (mU/l)	1.87 ± 0.33	2.19 ± 0.70^a^	0.29 ± 0.57^a^
Testosterone (nmol/l)	20.41 ± 6.50	24.06 ± 8.34	3.64 ± 3.80*
Cortisol (µmol/l)	0.18 ± 0.05	0.16 ± 0.03^a^	−0.02 ± 0.03^a^
Testosterone/cortisol (nmol/l/µmol/l)	132.97 ± 95.96	133.80 ± 29.76^a^	23.30 ± 26.70^a^

Data are presented as mean ± standard deviation

*BMI* body mass index; *FMI* fat mass index; *FFMI* fat free mass index; *MM* muscle mass; *EB* energy balance (according to body composition); *fT3* triiodothyronine; *fT4* thyroxine; *TSH* thyroid stimulating hormone

^*^Sign. difference between the measurement times *p* < 0.05, ** *p* < 0.01; ^a^*n* = 10

**Fig. 3:**
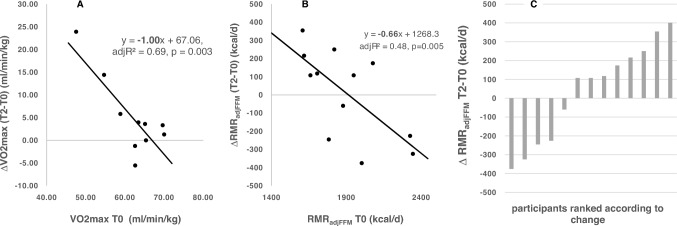
Regression analyses ∆T2T0-VO_2max_ on baseline (= T0) VO_2max_ (**A**), ∆T2T0RMR_adjFFM_ on baseline (T0) RMR_adjFFM_ (**B**) and inter-individual variance in exercise-related energy compensation of RMR in the group of ‘full completers’ (**C**; *n* = 13). See Fig. 2 and Table 3 for original data

Physical fitness before CST was negatively correlated with its increase in response to CST (Fig. 3A). While RMR_adjFFM_ before CST had a negative association with its decrease with CST (Fig. 3B), there was no significant association between CST-induced changes in VO_2max_ and CST-induced changes in RMR_adjFFM_. However, there was a considerable inter-individual variance in metabolic adaptation to CST (Fig. 3C).

### Is there a difference between successful and unsuccessful participants at the beginning of the training program?

Successful participants had a lower baseline (T0) body weight and higher energetic work efficiency at all levels of workload when compared with participants who dropped out at T1 (Table 2). Soldiers who were not found eligible for CST had a lower body weight, less physical fitness, and a lower energetic efficiency, while plasma levels of testosterone were slightly lower, and cortisol were slightly higher (Table 2).

### Is there a difference between successful participants and those who were not successful at the time of termination?

At T1, successful participants had a slightly higher T0–T1-decrease in BMI and a higher gain in VO_2max_ when compared with dropouts (Table 4). There were no differences in T1–T0-changes body composition, RMR or plasma hormone levels between the successful participants and those who dropped out. At T1, when compared to dropouts, work efficiency improved in completers compared to dropouts (Table 4). There were no between-group differences in the changes of plasma hormones (Table 4).

**Table 4:** T1T0-changes in body weight, body composition as well as metabolic and endocrine adaptations in ‘full completers’ compared to ‘exercise completers’

N = 28	∆Full completers	∆Exercise completers
Body weight and body composition		
Weight (kg)	−0.82 ± 1.27	−0.65 ± 1.56
BMI (kg/m^2^)	−0.25 ± 0.38*	−0.20 ± 0.47
FMI (kg/m^2^)	−0.21 ± 0.26	−0.21 ± 0.24
FFMI (kg/m^2^)	0.27 ± 1.14	0.30 ± 0.87
MM (kg)	0.42 ± 1.97	0.44 ± 1.43
EB (kcal/d)	−79 ± 308	−87 ± 253
Aerobic fitness		
VO_2max_ (ml/min)	230 ± 490^a^	−21.80 ± 453.84^b^
VO_2max_ (ml/min/kg)	3 ± 6^a^*	0 ± 6^b^
Resting energy expenditure		
REE (kcal/d)	−58 ± 206	−13 ± 241
REEadj (kcal/d)	−81 ± 221	−37 ± 297
REEpred (kcal/d)	−11 ± 17	−9 ± 21
REEm–REEp (kcal/d)	−47 ± 194	−4 ± 260
Respiratory exchange ratio	0.04 ± 0.11	0.05 ± 0.07
Work efficiency		
EE 10 km/h (kcal/min)	−0.70 ± 0.96^a^	−0.14 ± 1.90^b^
EE 10 km/h (kcal/min/kg)	−0.01 ± 0,01^a^	0.00 ± 0.03^b^
EE 10 km/h (kcal/min, adjFFM)	−0.56 ± 1.09^a^	0.18 ± 0.48^b^
EE 12 km/h (kcal/min)	−0.66 ± 0.87^a^	−0.37 ± 0.90^b^
EE 12 km/h (kcal/min/kg)	−0.01 ± 0.01^a^	0.00 ± 0.01^b^
EE 12 km/h (kcal/min, adjFFM)	−0.51 ± 1.00^a^	0.24 ± 0.65^b^
EE 14 km/h (kcal/min)	−0.28 ± 1.39^a^	−0.03 ± 0.86^b^
EE 14 km/h (kcal/min/kg)	0 ± 0.01^a^	0.00 ± 0.01^b^
EE 14 km/h (kcal/min, adjFFM)	0.13 ± 0.91^a^	0.26 ± 0.71^b^
EE 16 km/h (kcal/min)	0.29 ± 2.13^a^	0.00 ± 0.90^b^
EE 16 km/h (kcal/min/kg)	0.01 ± 0.03^a^	0.00 ± 0.01^b^
EE 16 km/h (kcal/min, adjFFM)	0.91 ± 2.58^a^	0.24 ± 0.65^b^
Hormones		
fT3 (ng/dl)	−0.01 ± 0.03	−0.02 ± 0.04
fT4 (ng/dl)	0.01 ± 0.06	−0.07 ± 0.11
TSH (mU/l)	0.26 ± 0.52	0.05 ± 0.70
Testosterone (nmol/l)	1.58 ± 3.89	1.13 ± 4.05
Cortisol (µmol/l)	−0.03 ± 0.06	0.01 ± 0.05
Testosterone/cortisol (nmol/l/µmol/l)	16.36 ± 60.06	−3.02 ± 70.44

Data are presented as mean ± standard deviation

*BMI* body mass index; *FMI* fat mass index; *FFMI* fat free mass index; *MM* muscle mass; *EB* energy balance (according to body composition); *VO*_*2max*_ maximum volume of oxygen consumed; *REE* resting energy expenditure; *REEadj* REE adjusted for FFM using regression analysis; *REEpred* predicted REE acc. to Harris and Benedict; *REEm − REEp* measured REE minus predicted REE acc. to Harris and Benedict; *EE* energy expenditure; (kcal/min, adjFFM): energy expenditure adjusted for FFM using regression analysis; *fT3* triiodothyronine; *fT4* thyroxine; *TSH* thyroid stimulating hormone; *sign. difference Full completers vs. Exercise completers, *p* < 0.05; ^a^*n* = 10; ^b^*n* = 12

## Discussion

### Is there a decrease in RMR in response to CST?

Although the soldiers were already in a good-to-excellent training condition before the start of the study protocol (as characterized by their VO_2max/kg_ compared to competitive athletes; see Marti et al. 1999, Knechtle 2002), as well as when compared to the average German population (i.e., 162% ± 18% of the 50th percentile and at 129% ± 12% of the 90th percentile above the physical fitness of the general German population; Finger et al. 2021), they showed a further improvement during CST with an average increase in VO_2max_ of 7% (Tables 1, 2 and Figs. 2A, 3A).

Contrary to the ‘constraint model’ of EE (Pontzer et al. 2016; Pontzer 2018; Urlacher et al. 2019; Careau et al. 2021), we did not observe an exercise-related change in RMR in response to our prolonged high-intensity exercise training protocol (Fig. 2B–D). Both the mean difference between measured and predicted RMR as well as the mean change in RMR adjusted for the change in FFM remained unchanged (Fig. 2C, D). These data may indicate that at the group level, there was no exercise-related energy compensation within the RMR component of TEE. Concomitantly, CST did not affect plasma testosterone and T3-levels, suggesting that there were no perturbations in two essential basal functions, i.e., the hypothalamic–pituitary–testicular axis and the hypothalamic–pituitary–thyroid axis (Table 3). By contrast, the increased work efficiency at low workloads shows that expending less activity-related energy is a metabolic compensation to CST in the non-resting energy expenditure component of TEE (Fig. 2E, F). This exercise-related compensation may reflect an improved mitochondrial function within skeletal muscle cells.

There was a considerable inter-individual variance in changes in RMR beyond the expected changes due to the decreases in FFM (Fig. 3C) which was associated with the mass-independent variation of RMR before CST: The higher basal RMR_adjFFM_ the greater was its response to CST (Fig. 3B). Obviously, ‘exercise-RMR-compensation’ occurs in some but not in all soldiers in response to CST (Fig. 3c). However, the metabolic basis of ‘compensators’ or ‘non compensators’ remains obscure. Based on our data, one may speculate that faced with the variance in RMR_adjFFM_ before CST soldiers who are at a lower range of their mass-independent metabolic rates are limited in the exercise-induced trade-off between resting and non-resting EE.

Data of a recent randomized controlled 24-week intervention study in formerly sedentary individuals who had a low cardiorespiratory fitness and were exposed to mild-to-moderate exercise volumes may add to our present findings (Flanagan et al. 2024). In that study, baseline TEE was negatively associated with exercise-related energy compensation which was explained by exercise efficiency rather than by basal metabolism*.* There was a considerable variance in the responses and approximately 50% of the study participants were considered as the so-called ‘compensators’. The authors speculated that individuals with a higher TEE may have a limited trade-off between the different components of TEE.

CST caused a variable and slightly negative energy balance resulting in a moderate loss in body weight and fat mass (Tables 3 and 4). With caloric restriction and weight loss, decreases in RMR are explained by a loss of FFM, changes in its anatomical composition and weight loss-associated hormonal adaptations, i.e., decreases in plasma levels of T3, leptin, insulin, as well as a reduced sympathetic nervous system activity, while plasma glucagon levels increased (Bosy-Westphal et al. 2013; Müller et al. 2015, 2021, 2023b). By contrast, in our CST-study, we did not find a similar hormonal pattern as observed at a slightly negative energy balance, which differed from the negative energy balance seen during semi-starvation (Müller et al. 2015, 2021) as well as during an extensive and prolonged US-transcontinental race (Thurber et al. 2019). Concomitantly, during CST, there was a minor loss in muscle mass only (Table 3).

When compared to previous data on the effects of caloric restriction without exercise, the lack of metabolic adaptation in RMR in response to CST may be explained by (i) the slightly negative energy balance only and/or (ii) a training effect which may counteract metabolic adaptation to a negative energy balance and/or (iii) some unmeasured behavioral adaptations to TEE (Broskey et al 2021). However, ‘energy constraint’ has been shown in weight stable subjects as well as in subjects who were in a negative energy balance (Pontzer et al 2012; Urlacher et al. 2019; Flanagan et al. 2024; McGrosky et al. 2024) questioning the idea that a negative energy balance is a mandatory requirement of a mass-independent decrease in RMR in response to exercise training. Although a constrained energy model suggests that energy expenditure, rather than energy balance, may influence essential physiological systems, training-associated changes in EE were also shown to be ‘additive’ (under conditions of positive energy balance) or ‘constrained’ (in response to a negative energy balance) (Willis et al. 2022), suggesting that the discussion is not yet over.

Placing our data in a broader context, the US Army had already conducted research on the effects of physical and psychological stress on soldiers of special forces following different protocols (Friedl et al. 2000; Hoyt and Friedl 2006; O’Leary et al 2024). These studies differed from our training protocol due to the extremely high intensity, stress load, and long duration of the training protocols which had been considered as survival training in some protocols. Consequently, in the US-studies, body fat had fallen below a minimum of about 4–5% of body weight during combat and survival training (Friedl et al. 1994). In addition, in that studies, FFM decreased together with decreases in plasma testosterone levels (down to castration levels) and triiodothyronine (to hypothyroid levels). These data reflect a considerable decrease in the ‘anabolic drive’ in response to an exhausting training protocol. Concomitantly, cortisol levels increased at the end of the protocol reflecting a high stress ‘load’ of the protocol (Friedl et al. 2000), which obviously differed from normal plasma cortisol levels seen after CST (Table 3). In these studies, detrimental changes in body composition are in part explained (i) by insufficient energy supply of special forces soldiers as well as (ii) by alimentary limits on sustained maximal human energy expenditure (Thurber et al. 2019).

Chronic energy deficiency is associated with negative health consequences, while physical capacity may be maintained due to reduced energy trade-offs to other traits, e.g., the immune system, stress response, and reproduction (Pontzer 2018). There are several reasons for an insufficient energy intake of soldiers undergoing a high workload, e.g., inadequate catering regulations, mealtimes that collide with training schedules, rigid catering structures in the army, and soldiers' lack of knowledge about adequate nutrition. It has been suggested that these nutrition- and metabolism-related issues result in a sustained negative energy balance and nutrition-related decreases in physical performance which is again associated with increased risks of illness and injuries (Tharion et al. 2004; O’Leary et al. 2024). The issue also applies to a relevant extent to mission conditions. On average, special forces have a 19% higher energy requirement than “normal” soldiers, which is generally not considered in the catering regulations (Tharion et al. 2005). It is known that a weight loss of > 10% and harmful levels of body energy stores (< 5% body fat) are associated with a negative impact on health and performance (Tharion et al. 2005). Even 10 years after the inadequate energy supply of special forces was discovered, an inadequate energy supply of maritime special forces was still recommended (Margolis et al. 2014).

### Do the metabolic and endocrine responses to CST allow a differentiation between successful and unsuccessful participants?

Studying soldiers before CST, there were minor differences in body composition and EE at higher workload between completers and dropouts (Table 2). However, when compared to participants of CST soldiers who were not found eligible showed a lower VO_2max_, a higher work efficiency in low-intensity exercise, and a tendency towards lower plasma testosterone levels (Table 2). In addition, when compared with ‘full completers’ ‘exercise completers’ showed no improvements in VO_2max_ and work efficiency at low intensity (Table 4) which is not a new finding.

### Strength and limitations

The study has some major strength related to a highly standardized training protocol, an optimal supervision, and care for the study participants, detailed body composition analysis by a gold standard method and the state-of-the-art assessment of RMR.

In addition, our study has some limitations.

- As only male applicants were eligible for training as combat-swimmers, the results refer to males only. When compared to men, women may experience greater physiological stress, they may be prone to metabolic disturbances associated with energy deficit and, thus, also to stress fractures during military training. Sex differences in endocrine and metabolic adaptation during a 44 wk arduous military training have been addressed only recently (O’Leary et al. 2024). In this study, men experienced greater energy deficits than women due to higher energy expenditure, which was not compensated for by increased energy intake. However, these energy deficits did not impact fat or lean mass or metabolic and endocrine function.
- We could not directly assess TEE as well as PA which was partly due to the conditions of the combat-swimmer training protocol. However, the assessment of RMR, RER, and energetic efficiency in response to different workloads have allowed the characterization of specific aspects of ‘exercise-related energy compensation’.
- At T2, the required number of 15 successful participants indicated by the initial power analysis was missed with 13 successful participants (Table 3), while at T1, 28 soldiers could be studied (Table 4). In our study, the number of participants also limits the usefulness of a detailed evaluation of ‘compensators’ and ‘non compensators’ to identify factors that predispose someone to ‘constraint’. It is worthwhile to mention that in the 2024 study of Flanagan et al., sample size calculations based on the 8 wks exercise revealed as few as 11 participants per group only.
- The duration of CST may be considered as too short to expect changes in metabolic adaptation which may take longer time periods, i.e., more than12-24 weeks (Pontzer 2018). However, as far as the effect of caloric restriction is concerned, metabolic adaptations in RMR can be seen within 3d of semi-starvation (Müller et al. 2015). It is our present working hypothesis that mass-independent decreases in RMR in response to a negative energy balance result from a mismatch between cellular energy supply (equivalent to reduced energy intake plus the increase in endogenous lipid mobilization) and TEE. Following that idea, we would not expect any decrease in RMR in response to an extensive training protocol as long as cellular energy supply matches TEE. Since however starvation and exercise differ with respect to the ‘ranking’ of organ/tissue systems involved (i.e., 1. brain—2. liver—3. adipose tissue/skeletal muscle in the case of caloric restriction vs. 1. skeletal muscle—2. liver—3. adipose tissue during exercise; see Müller et al. 2019), they differ in metabolic responses which limits an analogy between the two metabolic situations.

## Conclusion

In contrast to the ‘constraint model’ of TEE, we did not observe an exercise-related energy compensation of RMR in response to a highly standardized and extensive exercise training protocol in elite soldiers. We did, however, find an improvement in energy efficiency under low-intensity work loads that suggests an energy compensation within the AEE-component of TEE. Further research is needed to compare the effect of an extensive training protocol on TEE, RMR, and AEE at different energy balances, i.e., under isocaloric vs. hypocaloric vs. hypercaloric conditions. Data cannot be made available to any data repository because of limitations of the German Data Protection Law.

## Abbreviations

AEE: Activity energy expenditure
CST: Combat-swimmer training
EE: Energy expenditure
EP: Exercise preconditioning
FFM: Fat-free mass
FM: Fat mass
HR: Heart rate
IC: Indirect calorimetry
KSM: Kommando Spezialkräfte der Marine (German Navy’s Special Forces Command)
PA: Physical activity
QMR: Quantitative magnetic resonance method
RMR: Resting metabolic rate
RER: Respiratory exchange ratio
MM: Muscle mass
SSS: Specific skills and stress
TEE: Total Energy Expenditure
VCO_2_: Carbon dioxide production
VO_2_: Oxygen consumption
